# Bridging virtual and real learning: the role of digital literacy and metaverse perspectives in enhancing academic motivation in nursing education

**DOI:** 10.1186/s12912-025-03681-x

**Published:** 2025-08-25

**Authors:** Shaimaa Mohamed Amin, Mohamed Hussein Ramadan Atta, Aliaa Ezz Eldin Abd Elmoaty, Hossam Ali Ismail, Mohamed Hashem Kotp, Hassan Ahmed Awad Basyouny, Mohamed Ahmed Aly, Ahmed Abdelwahab Ibrahim El-Sayed

**Affiliations:** 1 Community Health Nursing Department, Faculty of Nursing, Damanhur University, Damanhur, Egypt; 2 Psychiatric and Mental-Health Nursing Department, Faculty of Nursing, Alexandria University, Alexandria, Egypt; 3 Nursing Administration Department, Faculty of Nursing, Helwan University, Cairo, Egypt; 4 Department of Nursing, College of Applied Medical Sciences, Prince Sattam Bin Abdulaziz University, Wadi Addwasir, Saudi Arabia; 5 Nursing Administration Department, Faculty of Nursing, Alexandria University, Alexandria, Egypt

**Keywords:** Academic motivation, Digital literacy, Metaverse, Nursing education, Immersive learning, Nursing students, Technology-enhanced learning, Structural equation modeling

## Abstract

**Background:**

Academic motivation is crucial in nursing education, as it directly impacts students’ engagement, learning outcomes, and professional development. With the rapid integration of digital technologies in education, digital literacy and immersive platforms, such as the metaverse, offer new avenues to enhance student motivation. However, limited research has explored how these factors interact to shape academic motivation among nursing students.

**Objective:**

To examine the relationship between digital literacy, metaverse perspectives, and academic motivation among undergraduate nursing students, and to assess the mediating role of digital literacy in this relationship.

**Design:**

The study employed a cross-sectional descriptive research design.

**Method:**

A total of 427 undergraduate nursing students from a faculty in Cairo, Egypt, were recruited using simple random sampling. Data were collected in a time estimated to be about three months from January 2025 to April 2025, through validated written Arabic versions of the Digital Literacy Scale, Metaverse Scale, and Academic Motivation Scale. Descriptive statistics, correlation analyses, and structural equation modeling were used to analyze the data.

**Results:**

Students reported moderate to high levels of academic motivation, digital literacy, and awareness of the metaverse. Significant positive correlations were found among the three variables. SEM revealed that metaverse perspectives significantly predicted digital literacy (β = 0.802), which in turn predicted academic motivation (β = 0.306). A partial mediating effect of digital literacy was identified between metaverse perspectives and academic motivation.

**Conclusion:**

Digital literacy and metaverse engagement are key drivers of academic motivation in nursing education.

**Implications:**

Nursing curricula should integrate immersive technologies and digital literacy training to foster sustained academic motivation and better prepare students for digitally driven healthcare environments.

**Clinical trial number:**

Not applicable.

## Introduction

Academic motivation is a critical factor in nursing education, shaping students’ engagement, persistence, and academic performance [1, 2]. With advancements in educational technologies, digital literacy, and virtual platforms like the metaverse offer innovative opportunities to enhance student motivation are offered. Digital literacy, the ability to navigate, evaluate, and create information using digital tools, is increasingly vital for academic and professional success [3]. Meanwhile, the metaverse, with its immersive and interactive features, enables experiential learning that can boost both intrinsic and extrinsic motivation among nursing students [4].

Research indicates that integrating digital tools and virtual environments into nursing curricula may improve student motivation and strengthen professional commitment [5, 6]. However, while studies have separately examined digital literacy and virtual learning, few have explored how these elements jointly impact academic motivation in nursing. Most existing research focuses either on digital competence or the educational potential of immersive platforms, without addressing their combined influence on motivational outcomes [2–4]. This lack of empirical evidence highlights a clear gap in the literature. Therefore, there is a pressing need to investigate how digital literacy and metaverse-based learning environments can work together to foster academic motivation, engagement, and academic success in nursing education.

## Background

In today’s rapidly evolving digital landscape, nurses must possess robust digital literacy and information and communication technology (ICT) skills to ensure the provision of safe, efficient, and high-quality care [7, 8]. Digital literacy refers to the ability to locate, evaluate critically, and share information using digital tools [9]. As healthcare becomes more digitalized, nursing education must equip students to function effectively in technologically advanced environments [10]. However, rising student enrolments and increasing diversity pose challenges for educators in addressing varied digital competence levels among learners [11]. Harerimana et al. (2022) found that first-year nursing students exhibited wide variability in basic computer skills and digital device usage [12]. Similarly, Li et al. (2025) emphasized that digital literacy is not merely technical but also involves social responsibility and continuous professional growth [13].

Countries like the USA, Canada, and the UK have integrated health informatics into nursing curricula [14, 15]. In Australia, the National Nursing and Midwifery Digital Health Capabilities Framework, launched in 2020, supports healthcare’s digital transformation [16]. The Australian Digital Health Agency (ADHA) earlier developed the 2018 Framework for Action, highlighting interoperability and safe data sharing. Aligning with this vision, the Australian Nursing and Midwifery Accreditation Committee (ANMAC) Standard 3.4 mandates that curricula incorporate contemporary and emerging technologies [17]. The Digital Health Capability Framework identifies five essential domains: Digital Professionalism, Leadership and Advocacy, Data and Information Quality, Information-Enabled Care, and Technology, all of which aim to prepare students for modern, person-centered healthcare [17].

Recent innovations have introduced immersive technologies like the metaverse into nursing education. A study by Lee et al. (2023) explored a mock trial in VRChat, revealing active student engagement and appreciation for immersive learning compared to traditional methods [18]. These virtual platforms enabled reflective and experiential learning, enhancing students’ empathy and understanding of diverse perspectives [18]. Incorporating immersive technologies like the metaverse not only enhances technical competence but also prepares students for future digital healthcare environments [19].

The metaverse—a digitally connected environment merging real and virtual spaces—has promising applications in education [20, 21]. Through personalized avatars, learners navigate immersive 3D environments, simulating real-life scenarios. Unlike conventional tools such as Zoom, which offer limited interaction, the metaverse enhances realism and presence, making learning more engaging and interactive—particularly relevant during remote education periods like the COVID-19 pandemic [22]. Given the digital fluency of today’s students, integrating the metaverse into nursing curricula aligns with their learning needs and future practice expectations [23].

Growing awareness and positive attitudes toward the metaverse are evident among nurses and students. Ergin et al. (2024) reported that 81.6% of nurses expressed confidence in using the metaverse for future patient education, and 46% believed they could participate in virtual nursing [24]. Sezer et al. (2024) also found that health sciences students, including nursing students, had high metaverse awareness and were receptive to adopting immersive technologies in their professional roles [25]. These findings suggest a readiness in both students and professionals to embrace immersive digital tools in healthcare, highlighting the importance of incorporating such innovations into nursing education [15].

Academic motivation plays a vital role in students’ academic success, persistence, and engagement [1, 4, 26, 27]. It affects attendance, performance, satisfaction, and use of metacognitive strategies. In nursing education, where students face emotional and clinical demands, motivation is essential for learning and professional resilience [28]. The Academic Motivation Scale, based on self-determination theory, assesses three types of intrinsic motivation (to know, to accomplish, to experience stimulation), three types of extrinsic motivation (external, introjected, identified regulation), and amotivation [29]. Given the global demand for skilled nurses and an aging population, maintaining high academic motivation is crucial for building a competent, future-ready workforce [3, 30].

Research shows a positive correlation between academic motivation and professional commitment among nursing students, and a negative relationship with academic burnout and depression [2, 31, 32]. Motivated students often demonstrate greater confidence and resilience, yet motivation can decline over time due to clinical pressures and academic challenges [33, 34]. Understanding these fluctuations is vital for sustaining engagement throughout nursing education [35].

This study explores how digital literacy and metaverse perspectives can enhance academic motivation in nursing. As healthcare and education rapidly embrace digital technologies, nursing educators need to understand how digital competence supports student learning and professional readiness [36]. Digital literacy equips students to access online resources, use virtual platforms, and build essential academic and clinical skills [3, 37]. The metaverse, with its immersive and experiential capabilities, presents a novel opportunity to engage learners in realistic clinical simulations, potentially boosting intrinsic motivation and conceptual understanding [4, 5].

While other fields have documented improved engagement and knowledge retention through virtual learning, this area remains underexplored in nursing. By investigating these relationships, this study aims to guide educators in adapting curricula for the digital age, enhancing both motivation and long-term professional commitment among nursing students [1, 2].

## Methods

### Research aim

This study aimed to assess the role of digital literacy and metaverse perspectives in enhancing academic motivation in nursing education.

### Research objectives

1. Assess the level of digital literacy among nursing students.
2. Assess nursing students’ perspectives and attitudes toward the use of metaverse technologies in education.
3. Assess the academic motivation levels among nursing students.
4. Examine the relationship between digital literacy, metaverse perspectives, and academic motivation.

### Research design

The study utilized a cross-sectional descriptive research design, adhering to the Strengthening the Reporting of Observational Studies in Epidemiology (STROBE) guidelines.

### Setting

The study was conducted at the Faculty of Nursing, Helwan University, in Cairo Governorate, Egypt. The Egyptian Ministry of Higher Education oversees the faculty and complies with national nursing education standards. The faculty consists of six specialized departments, each focusing on a different specialty of nursing. The undergraduate and postgraduate programs were designed on a credit-hour system, offering a well-structured method for monitoring academic progress and rigorously evaluating educational outcomes.

### Sample size and study participants

**Fig. 1:**
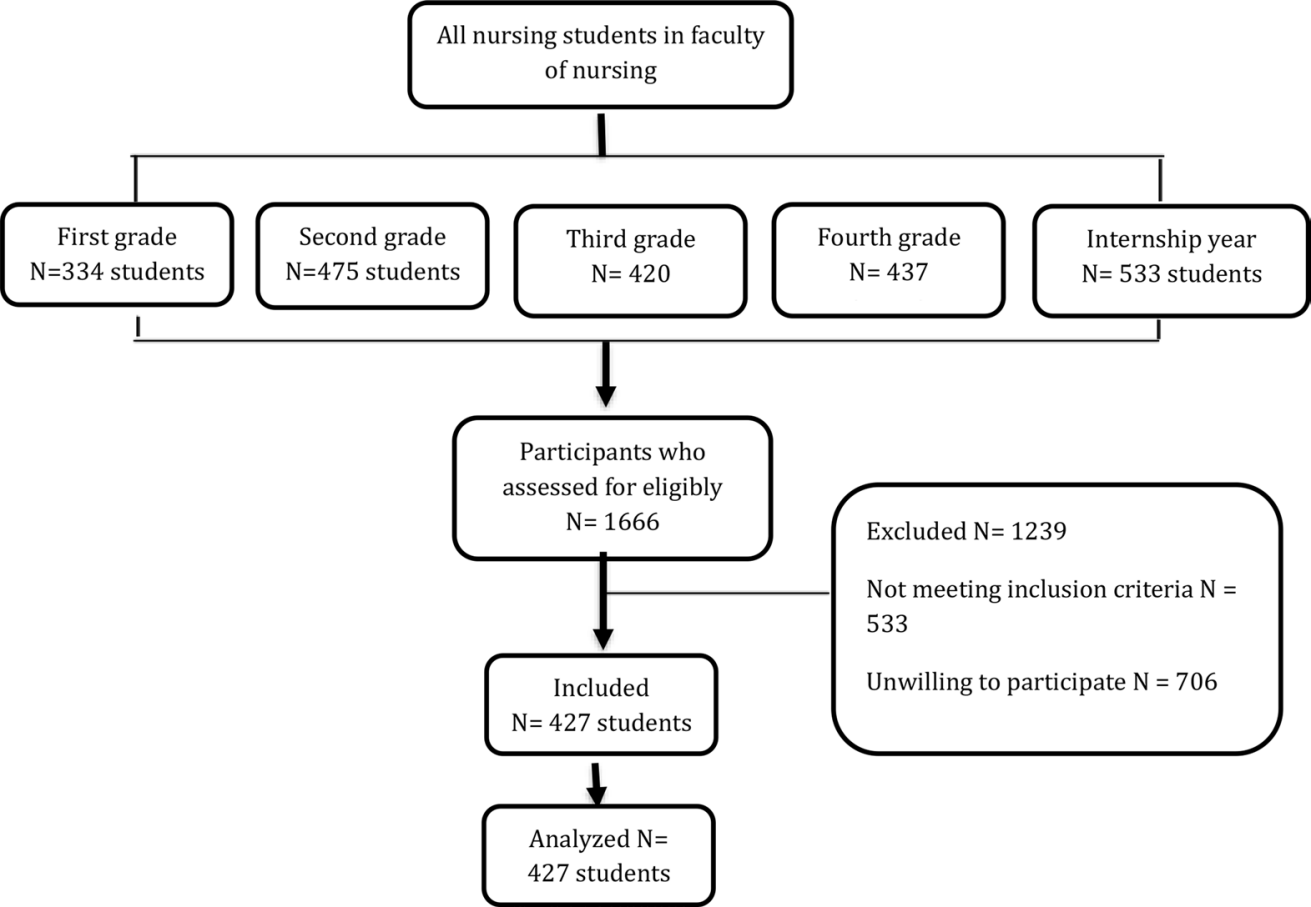
Shows the flow diagram of the included participants

The study applied a simple random sampling technique to select participants from the faculty of nursing. The participants of this study were described within the following inclusion criteria that they were undergraduate nursing students currently enrolled in the Faculty of Nursing for the academic year 2024–2025 who were accepted to participate in the study. Exclusion criteria included postgraduate students, students not enrolled in the nursing program, internship students, those with diagnosed mental health disorders that could affect their perception, and those who refused to participate in the study. A simple random sampling method was used to recruit participants who agreed to participate in the study, see Fig. 1. A total of 427 students were included.

### Sampling procedure

A simple random sampling was used to select students from the selected faculty of nursing. The sample size was estimated using Cochran’s formula (Cochran, 1977). The formula used in the equation seems to follow the standard form:

$$\:n=\frac{N\times\:P\left(1-P\right)\:}{\left[\right(\text{N}-1\left(\text{d}2\div\text{z}2\right)]+\text{p}(1-\text{p}\left)\right]}$$

Where:

- n = required sample size.
- N = population size.
- P = estimated proportion (0.5 for maximum variability).
- z = z-value corresponding to the desired confidence level (1.96 for 95%).
- d = margin of error (0.05).

Based on this formula, the final sample size was determined to be 427 students. Ensuring a confidence level of 95% and a maximum error margin of 5%.

### Measurements tools

#### Demographic form

The form consists of socio-demographic information, including age, sex, academic year, place of residence, family type, income, parents’ educational level, Access to technology, Internet access, used teaching methods, experience with online learning platforms, used virtual reality (VR) or augmented reality learning, and engagement with digital learning platforms.

### Digital literacy scale (DLS)

The DLS was developed by (Avinç & Doğan, 2024) [34] to measure the digital literacy competencies of nursing students, particularly in terms of their ability to access, evaluate, create, and communicate information using digital technologies. It assesses the foundational skills necessary for navigating virtual and digital learning environments, which are highly relevant in the context of blended or metaverse-enhanced education. Consists of 20 items, rated on a 4-point Likert scale: 1 = Strongly Disagree, 2 = Disagree, 3 = Agree, 4 = Strongly Agree. Total scores range from 20 to 80, with higher scores indicating higher levels of digital literacy. The scale demonstrates robust validity and reliability.

The internal consistency of the scale, measured by the computed α coefficient for the DLS questionnaire, stood at an impressive 0.982, indicative of a remarkably high level of internal consistency. Omega coefficient, which was found to be 0.89, reflecting a high level of reliability. Exploratory factor analysis confirmed the scale’s validity, showing factor loadings between 0.77 and 0.91. These factors collectively explained 71.54% of the total variance, with internal consistency remaining stable. The Kaiser–Meyer–Olkin (KMO) statistic yielded a value of 0.965, and the Bartlett test of sphericity exhibited statistical significance (χ2 = 6335.707, *df* = 190, *p* < 0.00). The current study reported the scale’s reliability coefficient as 0.93, further indicating excellent internal consistency. An exploratory factor analysis (EFA) was also conducted to revalidate the tool measurement following its translation into Arabic. Factor loadings ranged from 0.60 to 0.83 before rotation and improved to 0.68 to 0.91 after varimax rotation. It maintained excellent reliability after translating the scale into Arabic, with a Cronbach’s alpha of 0.97, affirming the scale’s strong construct validity and the suitability of the sample for factor analysis.

### Metaverse scale (MS)

The Metaverse Scale, developed by Özdemir et al. (2022) [35], was used to measure students’ awareness, knowledge, and attitudes toward the use of metaverse technologies in educational contexts. The scale assesses how nursing students perceive and are prepared to engage with immersive technologies within learning environments. It comprises four sub-dimensions: technology, digitalization, social, and lifestyle, and includes 15 items rated on a 5-point Likert scale (1 = Strongly Disagree to 5 = Strongly Agree). Total scores range from 15 to 75, with higher scores indicating greater awareness and more positive attitudes toward the metaverse. The scale demonstrated strong validity and reliability.

Exploratory Factor Analysis (EFA) revealed that the four-factor structure accounted for 55.17% of the total variance. Confirmatory Factor Analysis (CFA) confirmed the construct validity, with fit indices (CMIN/df, RMSEA, GFI, CFI, and SRMR) falling within acceptable ranges. The Cronbach’s alpha coefficient for the overall scale was 0.813, indicating good internal consistency. In the current study, after translation into Arabic, the scale maintained excellent reliability, with a Cronbach’s alpha of 0.82. Validity was confirmed through Exploratory Factor Analysis (EFA), which revealed factor loadings between 0.55 and 0.89 after varimax rotation. The Kaiser–Meyer–Olkin (KMO) measure was robust at 0.803, and Bartlett’s test of sphericity was highly significant (*p* ≤ 0.05), affirming the appropriateness of the data for factor analysis. All items on the scale were thus retained.

### Academic motivation scale (AMS-C-28)

The Academic Motivation Scale – College version (AMS-C-28), developed by Vallerand et al. (1992) [29], was used to assess the academic motivation of nursing students, encompassing both intrinsic and extrinsic motivation based on Self-Determination Theory. The scale evaluates the underlying reasons driving students to engage in academic tasks and how these motivations influence their engagement and persistence in education. It consists of 28 items distributed across three dimensions: intrinsic motivation (12 items), extrinsic motivation (12 items), and amotivation (4 items). Items are rated on a 5-point Likert scale (1 = Not at all, 2 = A little, 3 = Moderately, 4 = A lot, 5 = Exactly). Each subscale is scored separately, with scores ranging from 4 to 20, where higher scores indicate a stronger orientation toward that particular type of motivation. The scale has demonstrated high internal consistency, with Cronbach’s alpha values exceeding 0.80 across subscales. Confirmatory Factor Analysis (CFA) yielded a goodness-of-fit index of 0.89, and the Bartlett’s test of sphericity was statistically significant (χ² = 1228.27, df = 329, *p* < 0.001), supporting the scale’s construct validity. In the current study, the AMS-C-28 maintained excellent reliability following translation into Arabic, with a Cronbach’s alpha of 0.85, confirming its internal consistency and suitability for use in the target population.

In addition to analyzing individual subscale scores, total academic motivation was computed by summing all AMS-C-28 items. Furthermore, the Relative Autonomy Index (RAI) was calculated following the approach by Vallerand (1997) and Ryan & Deci (2020) using weighted means of the subscales to reflect overall autonomous motivation among students. The RAI formula was: (2 × IM) + (1 × Identified) – (1 × Introjected) – (2 × External Regulation) – (3 × Amotivation).

### Data collection procedures

Data for this study were collected in time estimated to be about three months from January 2025 to April 2025, following the acquisition of all necessary ethical and administrative approvals. Before data collection, the researchers clearly explained the objectives of the study to each student, emphasizing the voluntary nature of participation. Written and verbal informed consent was obtained from all participants. Students were assured of the anonymity and confidentiality of their responses. A simple random sampling technique was employed to recruit participants who consented to take part in the study. A complete list of student identification numbers was obtained from the Learning and Student Affairs Office. Questionnaires were administered in written form in quiet and comfortable settings, typically between sessions from 9:00 a.m. to 4:00 p.m., Sunday through Thursday.

The researchers personally met with each student to explain the purpose of the study and the procedure for completing the questionnaires. They remained present throughout the data collection process to provide clarification and ensure a distraction-free environment. On average, students required 10 to 15 min to complete each questionnaire, with the total time for all study tools ranging between 15 and 20 min. Completed questionnaires were reviewed immediately to ensure data completeness, and any missing information was addressed promptly.

### Ethical considerations

Ethical approval was obtained from the Ethical Committee of the Faculty of Nursing at Helwan University,number 46 on 25 Jan 2025. The study adhered to ethical principles outlined in the Helsinki Declaration, protecting participants’ rights and well-being. All participants provided written informed consent following a thorough explanation of the study’s objectives. Participants’ privacy and anonymity were rigorously protected, and all data collected was treated with the utmost confidentiality. Furthermore, participants were explicitly informed that they had the right to withdraw from the study at any time.

### Statistical design

Data were analyzed using SPSS version 26 and SmartPLS 4. Descriptive statistics—including means, standard deviations, frequencies, and ranges—were used to summarize the demographic data and baseline characteristics of the nursing students (*N* = 427) and to assess levels of academic motivation, digital literacy, and metaverse awareness. The normality of the collected data was thoroughly assessed before conducting the main statistical analyses. Both Shapiro-Wilk and Kolmogorov-Smirnov tests were applied to evaluate the distribution patterns of the primary study variables, including academic motivation, digital literacy, and metaverse perspectives. In addition, descriptive indicators of normality, such as skewness and kurtosis values, were calculated to further confirm the data distribution. The results indicated that all variables demonstrated acceptable levels of normality with skewness and kurtosis values falling within the acceptable threshold of ± 2. Furthermore, no significant violations of normality were detected based on the statistical tests (*p* > 0.05 for most variables).

Independent-samples t-tests and one-way ANOVA were conducted to examine differences in the three main variables based on students’ educational, technological, and sociodemographic characteristics. Pearson correlation analysis was performed to explore bivariate relationships among the key study constructs.

To examine the structural relationships and test the mediating role of digital literacy, Structural Equation Modeling (SEM) was employed. Model fit was evaluated using several indices, including χ²/df, GFI, AGFI, IFI, TLI, CFI, and RMSEA, all of which indicated a good model fit. Both direct and indirect effects were assessed through standardized path coefficients. A p-value of less than 0.05 was considered statistically significant for all analyses.

## Results

Table 1 presents the comparison of academic motivation, metaverse perspectives, and digital literacy among nursing students (*N* = 427) based on their sociodemographic characteristics. Significant differences were found in several variables. A number of variables showed notable variations. The academic motivation scores of students from extended families were significantly higher than those of students from nuclear families (*p* < 0.01). Academic motivation was also significantly correlated with the mother’s educational attainment (*p* < 0.01), with students who had mothers with higher education (secondary or university) demonstrating higher levels of motivation and digital literacy. Furthermore, there was a statistically significant correlation between father’s educational attainment and both academic motivation (*p* < 0.01) and metaverse perspectives (*p* < 0.01), favoring students whose fathers had completed higher education.

Table 2 examines how educational and technological factors affect nursing students’ (*N* = 427) digital literacy, metaverse perspectives, and academic motivation. The findings indicate a number of noteworthy correlations. Digital literacy (*p* < 0.01), metaverse engagement (*p* < 0.001), and academic motivation (*p* < 0.001) were all significantly higher among students with access to technology. Academic motivation (*p* < 0.01) and digital literacy (*p* < 0.01) were also significantly correlated with the type of internet access, with those who used mobile data scoring the highest and those who used university or no access scoring the lowest.

Students’ experiences with online learning platforms showed a particularly strong effect. Digital literacy, metaverse perspectives, and academic motivation were all significantly higher among advanced users than among beginners or intermediate users (all *p* < 0.001). Digital skills and motivation increased gradually from “never” to “always” users, and the frequency of using digital learning platforms had a significant effect on all three domains (all *p* < 0.001).

The three primary study variables’ mean scores and ranges are compiled in Table 3. Nursing students reported a moderate to high level of academic motivation (Mean = 95.10, SD = 19.94), with a full range of scores observed. With a mean score of 49.79 (SD = 8.58) on the metaverse scale. Among the three domains, the digital literacy scale had a mean = 70.87 (SD = 10.21).

Table 4 displays the Pearson correlation coefficients among the three main study variables. Metaverse perspectives and academic motivation were found to be significantly positively correlated (*r* = 0.575, *p* < 0.001). Likewise, there was a positive correlation between digital literacy and academic motivation (*r* = 0.570, *p* < 0.001). Digital literacy and metaverse perspectives showed the strongest correlation (*r* = 0.802, *p* < 0.001).

Table 5 presents the Pearson correlation coefficients between the Relative Autonomy Index (RAI) and the main study variables, including total academic motivation, metaverse perspectives, and digital literacy among nursing students. Results show a positive and statistically significant correlation between RAI and total academic motivation (*r* = 0.287), supporting its validity in reflecting general motivational tendencies. Correlations with metaverse perspectives and digital literacy were weaker, indicating that RAI primarily captures autonomous motivation rather than technological engagement or skill levels.

The findings of the structural equation modeling (SEM) study that looked at the connections between nursing students’ academic motivation, digital literacy, and metaverse perspectives are shown in Tables 6 and 7. All fit indices showed acceptable values for the model, as indicated in Table 4: χ²/df = 2.143, GFI = 0.991, AGFI = 0.965, IFI = 0.994, TLI = 0.982, CFI = 0.993, and RMSEA = 0.059. These indices show how well the suggested model matches the observed data.

Table 7 details the standardized path coefficients, showing both direct and indirect effects. Digital literacy was strongly influenced by the metaverse perspective (β = 0.802), and academic motivation was significantly predicted by digital literacy (β = 0.306). The metaverse also had an indirect effect through digital literacy (β = 0.245) and a direct effect on academic motivation (β = 0.330), for a total effect of 0.575.

**Fig. 2:**
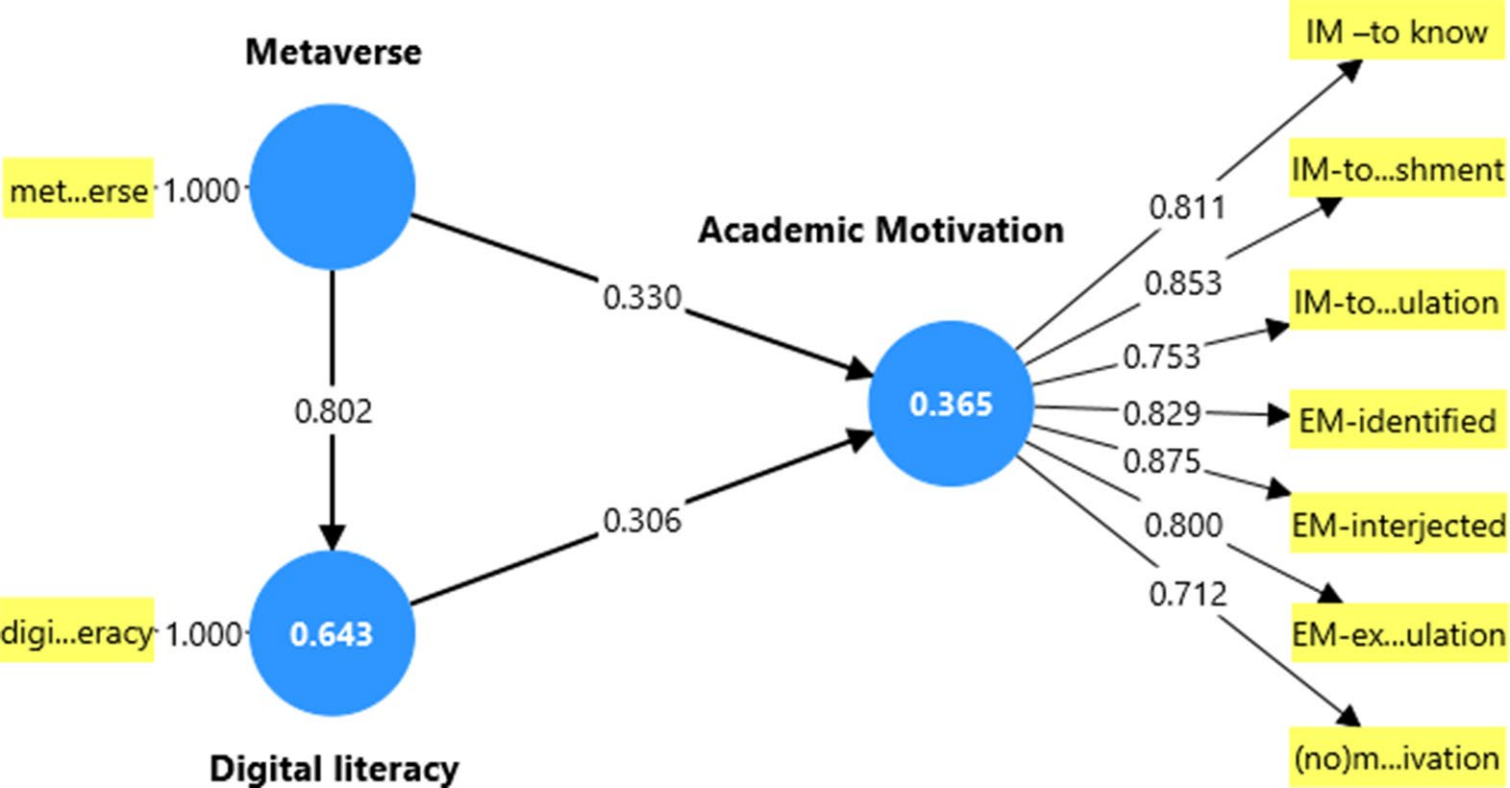
Structural equation model for the influence of metaverse and digital literacy on academic motivation

The structural equation model (SEM), along with its subdimensions, is depicted in Figure 2 as it examines the connections between academic motivation, digital literacy, and metaverse perspectives. According to the model, metaverse viewpoints directly improve academic motivation (β = 0.330) and have a greater direct impact on digital literacy (β = 0.802). Academic motivation is positively impacted by digital literacy as well (β = 0.306). These results lend credence to a partial mediation effect, in which academic motivation and metaverse perspectives are mediated by digital literacy.

Academic motivation is represented as a latent construct composed of seven observed variables: intrinsic motivation (to know, toward accomplishment, to experience stimulation), extrinsic motivation (identified, introjected, external regulation), and a motivation. A robust measurement model is indicated by the strong standardized loadings for all subdimensions, which range from 0.712 to 0.875. The proposed model’s theoretical underpinnings and structural integrity are supported by the fact that it explains 64.3% of the variance in digital literacy and 36.5% of the variance in academic motivation.

**Table 1:** Sociodemographic characteristics of nursing students (*N* = 427)

Variable	*N* (%)	Academic motivation (Mean ± SD)	Metaverse (Mean ± SD)	Digital literacy (Mean ± SD)
**Age**				
< 22	357 (83.6%)	94.88 ± 20.03	49.43 ± 10.61	67.52 ± 14.89
≥ 22	70 (16.4%)	96.19 ± 19.58	51.43 ± 11.76	67.84 ± 14.83
P value		0.613	0.191	0.866
**Sex**				
Male	188 (44.0%)	93.06 ± 22.05	49.40 ± 12.01	67.64 ± 15.73
Female	239 (56.0%)	96.69 ± 18.00	50.04 ± 9.79	67.51 ± 14.18
P value		0.068	0.556	0.933
**Occupation**:				
Working	91 (21.3%)	94.30 ± 24.72	50.32 ± 13.41	68.04 ± 17.14
Not working	336 (78.7%)	95.31 ± 18.47	49.61 ± 10.02	67.44 ± 14.21
P value		0.716	0.639	0.758
**Place of residence**:				
Rural	260 (60.9%)	94.84 ± 20.60	50.21 ± 11.19	67.96 ± 14.98
Urban	167 (39.1%)	95.49 ± 18.92	49.06 ± 10.21	66.96 ± 14.69
P value		0.739	0.274	0.499
**Family type**:				
Nuclear	157 (36.8%)	91.32 ± 21.34	48.60 ± 10.09	66.08 ± 13.08
Extended	270 (63.2%)	97.29 ± 18.78	50.44 ± 11.18	68.44 ± 15.76
P value		**<0.01**	0.082	0.097
**Family income**:				
Enough	252 (59.0%)	95.37 ± 20.46	50.05 ± 11.07	67.11 ± 14.88
Enough & save	110 (25.8%)	93.53 ± 18.96	49.76 ± 9.31	69.05 ± 13.58
Not Enough	65 (15.2%)	96.68 ± 19.64	48.65 ± 12.20	66.83 ± 16.81
P value		0.567	0.649	0.474
**Mother’s educational level**:				
Illiterate	56 (13.1%)	88.34 ± 25.95	47.59 ± 13.85	65.45 ± 18.21
Basic education	82 (19.2%)	94.63 ± 19.83	48.76 ± 10.31	66.91 ± 14.05
Secondary education	155 (36.3%)	97.05 ± 18.07	50.13 ± 9.98	68.08 ± 14.21
University & above	134 (31.4%)	95.94 ± 18.79	50.86 ± 10.55	68.26 ± 14.60
P value		**<0.01**	0.210	0.621
**Father’s Educational Level**				
Illiterate	39 (9.1%)	87.18 ± 24.32	44.41 ± 12.67	62.95 ± 17.88
Basic education	90 (21.1%)	92.83 ± 20.46	49.41 ± 10.66	66.69 ± 14.50
Secondary education	134 (31.4%)	97.47 ± 19.36	50.84 ± 10.63	68.80 ± 14.55
University & above	164 (38.4%)	96.28 ± 18.52	50.34 ± 10.29	68.15 ± 14.42
P value		**<0.01**	**<0.01**	0.154

**Table 2:** Comparison of academic motivation, metaverse perspectives, and digital literacy by technological and educational variables (*N* = 427)

Variable	*N* (%)	Academic motivation (Mean ± SD)	Metaverse (Mean ± SD)	Digital literacy (Mean ± SD)
**Access to technology**				
Yes	379 (88.8%)	96.33 ± 19.53	50.37 ± 10.71	68.20 ± 15.02
No	48 (11.2%)	85.35 ± 20.65	44.94 ± 10.58	62.60 ± 12.65
P value		**<0.001**	**<0.001**	**<0.01**
**Internet access**:				
At home	268 (62.8%)	93.49 ± 19.60	49.00 ± 11.12	66.56 ± 15.13
University /college	20 (4.7%)	87.05 ± 24.64	47.15 ± 11.09	62.25 ± 17.64
No access	2 (0.5%)	103.50 ± 27.58	60.00 ± 21.21	85.50 ± 20.51
Mobile data	137 (32.1%)	99.30 ± 19.18	51.47 ± 9.84	70.05 ± 13.36
P value		**<0.01**	0.052	<0.01
**Which teaching methods are currently used in your institution?**				
Traditional lectures	277 (64.9%)	94.98 ± 19.17	49.28 ± 10.86	66.60 ± 14.64
Hybrid learning	75 (17.6%)	95.93 ± 21.95	51.11 ± 9.99	69.45 ± 14.54
E-learning	75 (17.6%)	94.68 ± 20.88	50.20 ± 11.45	69.27 ± 15.83
P value		0.917	0.400	0.186
**Experience with online learning platforms**:				
Beginner	84 (19.7%)	83.61 ± 21.86	44.52 ± 11.75	59.98 ± 16.30
Intermediate	236 (55.3%)	95.08 ± 17.70	50.13 ± 9.54	68.07 ± 12.51
Advanced	107 (25.1%)	104.14 ± 18.50	53.06 ± 11.29	72.42 ± 16.20
P value		**<0.001**	**<0.001**	**<0.001**
**Have you ever used virtual reality (VR) or augmented reality (AR) for educational purposes?**				
Yes	151 (35.4%)	93.65 ± 21.50	50.54 ± 10.73	68.36 ± 15.60
No	276 (64.6%)	95.89 ± 19.03	49.34 ± 10.86	67.14 ± 14.45
P value		0.285	0.272	0.429
**How frequently do you engage with digital learning platforms (e.g.**,** simulations**,** virtual labs**,** online courses)?**				
Never	50 (11.7%)	83.12 ± 27.79	43.02 ± 12.91	61.22 ± 16.19
Rarely	74 (17.3%)	89.26 ± 19.53	48.59 ± 10.15	64.57 ± 13.89
Sometimes	205 (48.0%)	96.43 ± 15.46	49.84 ± 8.89	67.80 ± 13.52
Often	77 (18.0%)	102.48 ± 19.96	53.34 ± 12.51	71.70 ± 15.92
Always	21 (4.9%)	104.10 ± 20.90	56.05 ± 9.86	75.86 ± 15.98
P value		**<0.001**	**<0.001**	**<0.001**

**Table 3:** Descriptive statistics of academic motivation, metaverse perspectives, and digital literacy among nursing students (*N* = 427)

Variable	(Mean ± SD)	Min – Max
**Academic motivation**	95.10 ± 19.94	28–140
IM – to know	13.64 ± 3.46	4–20
IM – towards accomplishment	13.87 ± 3.50	4–20
IM – to experience stimulation	12.90 ± 3.47	4–20
EM – identified	13.75 ± 3.75	4–20
EM – introjected	12.41 ± 3.61	4–20
EM – external regulation	13.29 ± 3.71	4–20
A motivation	11.23 ± 4.09	4–20
**Metaverse**	49.79 ± 8.58	18–69
**Digital literacy**	70.87 ± 10.21	33–98

IM: Intrinsic Motivation EM- External regulation

**Table 4:** Correlation matrix between academic motivation, metaverse, and digital literacy (*n* = 427)

Variable	Academic motivation	Metaverse	Digital literacy
	*r*(*p*)	*r*(*p*)	*r*(*p*)
Academic motivation	1	0.575 (p = <0.001)	0.570 (p = <0.001)
Metaverse	0.575 (p = <0.001)	1	0.802 (p = <0.001)
Digital literacy	0.570 (p = <0.001)	0.802 (p = <0.001)	1

**Table 5:** Correlation matrix for RAI and main study variables (*n* = 427)

Variable	RAI	Total Motivation	Metaverse Perspective	Digital Literacy
RAI	1.000	0.287	0.115	0.086
Total Motivation	0.287	1.000	0.575	0.570
Metaverse Perspective	0.115	0.575	1.000	0.802
Digital Literacy	0.086	0.570	0.802	1.000

**Table 6:** Structure equation modeling fit indices

Fitness	χ²/df	GFI	AGFI	IFI	TLI	CFI	RMSEA
Acceptable Values	< 3	> 0.9	> 0.9	> 0.9	> 0.9	> 0.9	< 0.08
Mediation Model	2.143	0.991	0.965	0.994	0.982	0.993	0.059

GFI— Goodness of Fit Index; AGFI— Adjusted Goodness of Fit Index; IFI— Incremental Fit Index; TLI— Tucker-Lewis Index; CFI— Comparative Fit Index; RMSEA— Root Mean Square Error of Approximation

**Table 7:** Standardized direct, indirect, and total effects from SEM

Independent variable	Dependent variable	Direct effect	Indirect effect	Total effect
Metaverse	Digital Literacy	0.802		0.802
Digital Literacy	Academic Motivation	0.306		0.306
Metaverse	Academic Motivation	0.330	0.245	0.575

## Discussion

Digital literacy and metaverse perspectives play a synergistic role in enhancing academic motivation among nursing students. The metaverse and digital literacy promote positive attitudes towards learning by providing nursing students with new learning opportunities that are exciting, engaging, and interactive, suggesting that environments are enriching the learning process that enhance academic motivation and achievement [38]. So, this study aimed to assess the role of digital literacy and metaverse perspectives in enhancing academic motivation in nursing education.

The current study highlights the influence of demographic, socioeconomic, and academic factors on students’ perceptions and attitudes toward digital literacy, metaverse perspectives, and academic motivation. The academic motivation scores of students from extended families were significantly higher than those of students from nuclear families. Academic motivation was also significantly correlated with the mother’s educational attainment, with students who had mothers with higher education (secondary or university) demonstrating higher levels of motivation and digital literacy. Furthermore, there was a statistically significant correlation between father’s educational attainment and both academic motivation and metaverse perspectives, favoring students whose fathers had completed higher education.

These findings are consistent with Riego de Dios and Guevara (2024) [39]. who suggest that the extended family may offer certain motivational advantages over the nuclear family. Similarly, Häfner et al. (2018) [40] confirmed that the quality of family relationships-characterized by cohesion, support, and positive communication-emerges as a critical predictor of student learning motivation. Supportive extended families tend to foster higher intrinsic motivation and academic performance. Furthermore, Adikwu (2018) [41], who conducted a study in North Central Nigeria, agreed that Parental education, especially that of mothers, is consistently linked to higher academic motivation and digital literacy in students. Students whose mothers have attained secondary or university education tend to display greater motivation and digital skills, indicating that the educational background of both parents shapes not only motivation but also students’ readiness for modern learning environments.

Our study findings may be due to extended families that often provide a larger network of support, including emotional, financial, and academic assistance, where more adults in the household can serve as positive role models, emphasizing the importance of education and shared responsibilities within the family, which may instill a sense of duty and achievement in students. Also, educated mothers are more likely to instill a strong value for education in their children so that they may be better equipped to provide academic assistance and guidance. Those who are more likely to be digitally literate themselves can positively influence their children’s digital skills. Similar to Mothers, educated fathers also contribute to a household culture that values education. Educated fathers may be more open to and knowledgeable about emerging technologies, influencing their children’s attitudes as well. They are more likely to understand how the metaverse can positively impact their futures.

Our findings revealed that several noteworthy correlations, for instance, digital literacy, metaverse engagement, and academic motivation, were all significantly higher among students with access to technology. Furthermore, Academic motivation and digital literacy were also significantly correlated with the type of internet access, with those who used mobile data scoring the highest and those who used university or no access scoring the lowest. These findings are consistent with other studies that have explored students’ perceptions about digital literacy, metaverse perspectives and academic motivation, For example, the two studies which conducted by Zaidi et al., (2024) and Jeon et al., (2024) [42, 43] on nursing students with similar characteristics highlight the technological readiness and availability of access to technology for both educators and students can support immersive metaverse experiences or create disparities in access to metaverse-based learning, particularly for students from underprivileged backgrounds or institutions with limited resources.

Similarly, research conducted by Lee et al. [44], at two different universities in South Korea, indicated that the metaverse technology facilitates learning engagement and collaboration among students through effective communication capabilities resulting from social belonging and collaborative learning, which can affect and have a good impact on academic motivation. A similar study by Harerimana et al., 2022 [11] conducted at a university in South Africa found that 89.3% of undergraduate nursing students used their smartphones and 78.7% used laptops, and only 22% used university desktops.

Additionally, the study that conducted by Ata et al., (2019) [45] In Turkey reported that regarding digital literacy in university, students who were competent in digitalization and the technology field make efficient use of both the Internet and digital media. On the same line Vu et al., (2022) [46] Stated that the adoption of technology, the internet, and effective learning methods will lead to higher levels of achievement, which in turn will lead to higher academic motivation.

The current findings present that students’ experiences with online learning platforms showed a particularly strong effect. Digital literacy, metaverse perspectives, and academic motivation were all significantly higher among advanced users than among beginners or intermediate users. The level of digital skills and motivation increased gradually, and the frequency of using digital learning platforms had a significant effect on all three domains. These findings align with the study conducted in Jordan by Al-Nawaiseh et al. (2023) [47], who suggested that students who have advanced skills, engaged in a digital learning experience, and used reality simulation had high levels of acquisition and retention of information, which ultimately leads to higher motivation and thus academic achievement.

Similarly, our results are in agreement with a study conducted by Çetinkaya et al. (2024) [38] At a state university in Turkey which demonstrated that students who were digital natives accustomed to learning via technology, more motivated and active educated which can be maintained by utilizing the advantages of metaverse-based education, thus increasing academic motivation, so, metaverse is an effective educational tool that can facilitate students’ learning and increase motivation more effectively than traditional face-to-face instruction or e-learning using digital learning platforms.

Throughout our study, we assessed the nursing students’ levels of digital literacy, metaverse, and academic motivation. They reported a moderate to high level of academic motivation, digital literacy, and metaverse. These results coincide with those presented among nursing students in the study conducted by Al-Salman and Haider (2021) [48] In Jordan on 4,037 undergraduate students were found that students have higher levels of digital literacy, better academic performance, and better scores on clinical evaluations. Also, supported by Ibrahim et al. (2023) [49] Studies conducted in the United Arab Emirates show that nursing students have higher levels of digital competence and internet skills that mirror their enthusiasm to achieve more in academic learning, including higher exam scores and better grades.

Similarly, this finding was confirmed by Sirakaya et al. (2018) [50], and Cai et al. (2019) [51] Those who indicate that higher levels of use of metaverse environments can enhance the role of students in developing their digital skills. This indicates that virtual and augmented reality environments encourage students to learn in an atmosphere full of enthusiasm and participation. On the same line, Sezer et al., (2024) [25]Depicted that the mean scores of the students who obtained scores from the overall Metaverse Scale were 53 out of 75 points. This result can be interpreted as the participants’ knowledge and awareness levels regarding the Metaverse were high.

These results may be due to metaverse is a social communication framework for students which integrates learning experiences through the social communication paradigm and immerses themselves in a virtual technological society that interacts with the learning environment, effectively leading to enhancement of academic motivation and learning achievement.

As regards the findings of our study, structure equation modeling (SEM) underscores the correlation between study variables, for instance, metaverse perspectives and academic motivation, which was found to be significantly positively correlated. Likewise, there was a positive correlation between digital literacy and academic motivation. Digital literacy and metaverse perspectives showed the strongest correlation.

These study findings are consistent with Sungkawadee and Sittiwong (2024) [52], who reported in their study conducted at Naresuan University in Thailand that students’ learning achievements through the development of a metaverse learning ecosystem can promote self-directed learning, resulting in academic motivation for them, which was also statistically significant at the 0.05 level. Likewise, Students learning through the digital literacy environment for developing a virtual world classroom, Metaverse, have promoted the ability to think critically and self-directed learning of higher education students. Furthermore, Lilian et al. (2022) [53], who conducted a descriptive correlational research design at seven private institutions in Malaysia’s central area, reported that structural equation modeling of 583 students showed a positive relationship between learning motivational strategies and digital literacy.

Based on these findings, it is critical for educational institutions to equip their students with suitable technological infrastructures and digital resources to connect their learning with digital learning environments, resulting in good digital education that mirrors the academic motivation, because there is a demand to be involved earlier in the digital world. The findings of our study highlight the effect of study variables on each other. Digital literacy was strongly influenced by the metaverse perspective, and academic motivation was significantly predicted by digital literacy. The metaverse also had an indirect effect through digital literacy and had a direct effect on academic motivation, for a total effect of 0.575. Similarly, empirical research conducted by Al Yakin et al. (2023) [54] Supposed that digital literacy and level of technological competences have an impact on academic performance through learning motivation mediation, and the presence of significant effects of the metaverse on engagement and learning motivation.

The aforementioned findings may be due to in the metaverse, learners can explore their virtual environment, make choices, chart learning routes, and engage in self-directed activities. These environments can enable students’ autonomy to take pride in their work, increase intrinsic motivation, engagement, and enjoyment.

### Implications of the study

The findings of this study underscore the urgent need to embed digital literacy and metaverse-based learning within nursing education to foster greater academic motivation and engagement. Nursing educators should integrate structured digital literacy training into the curriculum, not as optional content but as a core competency aligned with 21st-century clinical practice. This includes teaching students how to critically evaluate digital information, interact within virtual learning environments, and apply these skills in patient care scenarios. Furthermore, metaverse platforms offer immersive, experiential learning opportunities that traditional classroom or 2D online methods cannot replicate. Incorporating virtual simulations, interactive avatars, and real-time clinical scenarios can help develop students’ confidence, decision-making, and reflective practice while stimulating both intrinsic and extrinsic motivation. The evidence from this study also points to the importance of tailoring digital and immersive learning experiences based on students’ existing digital competence, ensuring that beginner, intermediate, and advanced users receive targeted support. Ultimately, adopting these strategies will not only improve academic outcomes but will also prepare nursing graduates for technologically evolving healthcare environments.

At the leadership and policy level, this study calls for a strategic transformation in how nursing institutions and stakeholders approach educational innovation. Institutional leaders must invest in technological infrastructures such as VR headsets, high-speed internet, and immersive software, required to implement metaverse learning. Additionally, leadership must prioritize professional development by equipping faculty with the pedagogical and technical skills needed to integrate digital and immersive technologies effectively. At the policy level, accreditation bodies and ministries of education should revise national nursing education standards to include digital literacy and immersive simulation as essential learning outcomes. Moreover, equity considerations are critical: leaders must ensure that all students—regardless of socioeconomic status—have consistent access to the necessary tools and platforms to engage in digital learning. This may include creating institutional lending programs or subsidies for devices and internet access. Finally, broader implications extend to the healthcare sector, where digitally literate and metaverse-trained nurses can contribute to safer, more efficient, and technologically adept patient care.

### Limitations and future directions

This study has several limitations that warrant consideration. The evolving and loosely defined nature of the metaverse may affect how consistently students perceive and engage with it, potentially limiting the generalizability of findings. Contextual factors, such as cultural attitudes, institutional support, and disparities in technological access, may also influence outcomes and reduce applicability to other settings. Additionally, while validated tools were used, both digital literacy and academic motivation are multifaceted constructs influenced by unmeasured variables. The novelty of the metaverse may have introduced enthusiasm bias, temporarily inflating motivation levels. Finally, self-reported measures may not fully capture the dynamic and situational nature of academic motivation. Future research should explore longitudinal impacts of metaverse integration, incorporate objective performance metrics, and examine diverse cultural and institutional contexts to enhance generalizability and deepen understanding of how immersive technologies sustainably influence motivation and learning outcomes.

## Conclusion

This study underscores the transformative potential of digital literacy and metaverse technologies in fostering academic motivation among nursing students. Beyond enhancing engagement, these tools reflect a paradigm shift in how knowledge is constructed and internalized in healthcare education. As nursing moves toward a more digitalized future, intentional integration of immersive technologies must be coupled with pedagogical innovation, ensuring that motivation is sustained, learning is meaningful, and students are empowered for lifelong digital competence.

## Data Availability

The datasets and materials of the current study are available from the corresponding author upon reasonable request.
